# Medical Staff of the United States Army

**Published:** 1847-12

**Authors:** 


2. *Medical Staff of the United States Army.*—The medical corps of the army has gained unfading laurels, not only by devotion and courage in the strict line of their profession sician, that the result would be almost immediate death, the idea took with his friends, and he was bled by the doctor who suggested the practice. As might have been expected, in about six hours he was a corpse, and the great consolation seemed to be that he died so easily! Verily, on becoming acquainted with such practice, one would be tempted to believe that the Emperor Nero must have been a very tender-hearted man in condemning Seneca to so pleasant a mode of terminating his existence as bleeding to death. For the particulars see the Annals of Tacitus, Book 15, Sec. 60.

but also, when occasion offered, of using the sword as well as the amputating knife. Among many instances of great gallantry, we may mention that of Assistant Surgeon Roberts, who, in the thickest of the fight at the storming of Molino del Rey, meeting with a company, all of whose officers had been cut down, placed himself at its head, and received his death wound whilst leading the men to victory. —*Medical News and Library*.

